# Supplementary material for: Resolving the full spectrum of human genome variation using Linked-Reads
Source: Genome Res. 2019 Apr;29(4):635–45. doi: 10.1101/gr.234443.118 (PMC6442396; doi:10.1101/gr.234443.118)
Supplement: Supplemental Material [file supp_gr.234443.118_Supplemental_longranger-2.2.zip › longranger-2.2.tar.bz2!/lr2.2-public/lib/stan/cmdstan/stan/doc/api/html/functions_func_f.html]

Stan: Class Members - Functions


|  |  |
| --- | --- |
|  | Stan  2.5.0  probability, sampling & optimization |


- Main Page
- Related Pages
- Namespaces
- Classes
- Files

- Class List
- Class Index
- Class Hierarchy
- Class Members

- All
- Functions
- Variables
- Typedefs
- Enumerator
- Related Functions

- a
- b
- c
- d
- e
- f
- g
- h
- i
- j
- k
- l
- m
- n
- o
- p
- q
- r
- s
- t
- u
- v
- w
- x
- z
- ~


All Classes Namespaces Files Functions Variables Typedefs Enumerations Enumerator Friends Macros Pages

### - f -

- fast\_matrix\_copy\_()
  : stan::mcmc::ps\_point
- fast\_vector\_copy\_()
  : stan::mcmc::ps\_point
- fevals()
  : stan::optimization::ModelAdaptor< M >
- filtered\_values()
  : stan::common::recorder::filtered\_values< InternalVector >
- find\_arg()
  : stan::gm::argument
  , stan::gm::categorical\_argument
  , stan::gm::list\_argument
  , stan::gm::singleton\_argument< T >
- fixed\_param\_sampler()
  : stan::mcmc::fixed\_param\_sampler
- for\_statement()
  : stan::gm::for\_statement
- free\_all()
  : stan::memory::stack\_alloc
- fun()
  : stan::gm::fun
- fun\_exists()
  : stan::gm::add\_function\_signature
- function\_args()
  : stan::gm::generate\_init\_visgen
- function\_decl\_def()
  : stan::gm::function\_decl\_def
- function\_decl\_defs()
  : stan::gm::function\_decl\_defs
- functions\_grammar()
  : stan::gm::functions\_grammar< Iterator >
- fvar()
  : stan::agrad::fvar< T >


---

[ Stan Home Page ]

*© 2011–2014,
Stan Development Team.*
